# Chinese Doctors and Medical Treatment

**Published:** 1904-10

**Authors:** J. F. Bishop


### BUFFALO MEDICAL JOURNAL.

Vol. XLIV.—Lx. OCTOBER, 1904. No. 3

### ORIGINAL COMMUNICATIONS.

### Chinese Doctors and Medical Treatment.

By MRS. J. F. BISHOP, F. R. G. S.

THERE are many horrors and barbarities, coupled with much gross ignorance and superstition, to be found in Chinese medicine. Its theories have an extravagant and fantastic basis. Nothing sound can be built upon them, and at the back of all lies a belief in malignant demons as the source of disease tending, as is usual in the East, to exorcisms and incantations as a last resort.

But while sketching a system which on the whole merits to be supplanted, as much on the ground of chicanery and fraud with which it is interwoven as for more obvious reasons, I must not be understood as condemning the whole practice of Chinese medicine, for as men are often better than their creeds, so, often, is the practice of the Chinese doctor better than his theory. For instance, he knows the virtues of many of the valuable native herbs, especially in fevers; he realises the worth of counter irritants, and in some cases even uses the cautery successfully. He knows to some extent Chinese constitutions and what they will bear, and it may at least be said of him that, however bad his system is, it has not arrested the increase of his race. If he would drop lies and humbug, parts of his treatment are not reprehensive.

China claims to have a science of medicine dating from the haziest antiquity and medical works dating from a time long antecedent to the Christian era. A book in twenty-four volumes on "Internal Diseases and the Practice of Amputations,"[1] was written before the dawn of authentic history, and a work on eighty "Difficult or Doubtful Medical Questions," was completed in the third century, A. D., on which eleven commentaries were

1. At the present time Chinese doctors rarely, if ever, amputate, and unless they have had foreign instruction do not know how to tie an artery.

written before the fourteenth. A noted court physician published a work on the "Pulse" in A. D. 290, which has gone through many subsequent editions, and is greatly valued. A work on the "Eye and Its Diseases," appeared in the tenth century. A six volume work on fevers followed, which was succeeded in the thirteenth century by one on the "Diseases of Women," in twenty-four volumes, which appears at intervals even now in abridged editions. In the same century a twelve-volume treatise on fevers "came out." In 1340 a work in twenty volumes appeared, taking the wide range of the "Diseases of the Large and Small Bloodvessels, Nervous Diseases, Midwifery and Women's Diseases, Diseases of the Mouth, Teeth, and Throat, and the Treatment of Fractures and Arrow-wounds." About 1360, works were written by two doctors of renown on fevers and skin diseases, and apoplexy followed a little later.

The *opus magnum* of Chinese medical literature is, however, a highly esteemed work written by Chu Su, an Imperial Prince of the Ming dynasty, in one hundred and sixty volumes, containing two thousand lectures on about the same number of subjects, two hundred and forty diagrams, and about twenty-two thousand prescriptions. A famous "Materia Medica," in fifty-two books was compiled in the sixteenth century, from the works of eight hundred authors, but it only gives eighteen hundred and ninety prescriptions. In 1602 a work in one hundred and twenty volumes on "Fevers, Ulcers, and the Diseases of Women and Children," was published, succeeding an important one on hygiene in 1591. The latter treats of diet, drink, regimen, amusements, rest, study, proper clothing and how to prevent disease and live virtuously.

About the same time several short treatises on "Children's Diseases," with rules for their treatment appeared, together with a work on "Acupuncture," with a number of diagrams, in seven volumes. In 1674 an important work in eight volumes on the "Diseases of Maternity" was published and in 1684 another on the same subject, to which was added the management of children. Six volumes on eye diseases, several works on smallpox and two on cholera were published during the period of the Ming dynasty. In the latter half of the sevententh century famous works on the properties of drugs and on saving life in cases of attempted suicide and accident, were published. The most complete of modern Chinese works on "The Practice of Medicine," in ninety volumes, was put forward in 1740. It contains many plates and diagrams, and a praiseworthy and rigorous attempt to classify diseases.

The late Dr. Henderson, of Shanghai, and Dr. Hobson, to whom I am largely indebted give very lengthy lists of Chinese medical works. The above are only a few of the most celebrated.

Besides these renowned works there are a number of general and special treatises which carry much weight in China, all showing that whatever the quality may be, quantity is not lacking.

As to the quality those Englishmen and Germans who have studied Chinese medical literature testify unanimously that on the whole it is inconceivably deplorable. They have some anatomical diagrams, it is true, but so competent an authority as the late Dr. Lockhart says of these:

> They are just as if some person had seen an imperfect dissection of the interior of a human body, and then had sketched from memory a representation of the organs, filling up parts that were obscure out of his own imagination, and portraying what, according to his own opinion, the parts ought to be, rather than what in reality they are.

Dr. Henderson sums up the worthlessness of this great body of professedly scientific literature thus:

> In all their writings there is no evidence of disinterested industry, or yearning after knowledge or more light—their best theories are based upon empty speculations and wild fancy—in their endeavors to support what they consider consistency and harmony in their system of physics, they sacrifice not only truth, but also intelligibility and reason. In many of their writings their aim seems to be to make every subject as mysterious as possible, professing to admire and reverence most that which is least known and understood. In none of their works is there any evidence that human dissection was ever practised, so that both human and comparative anatomy are utterly unknown.—*Royal Asiatic Society's Journal,* North China, 1864.

Chinese medical science makes no distinction between veins and arteries, nor, consequently between venous and arterial blood. Of the functions of the brain, heart, lungs, liver and kidneys, the doctors know literally nothing. They believe that the human soul resides in the liver, and from this organ emanate all great and noble purposes.[1] The gallbladder plays very important part in their theories. It is the seat of courage; its size determines the boldness or timidity of any character, and its ascension in the body is the cause of anger. As the Chinese eat stag and rhinoceros horn and the dried blood of tigers to increase their courage, so they sometimes procure the gallbladders of bears, tigers, and

1. May the expression "white livered," to describe cowardice, be traced to the same belief? (This is undoubtedly true, as the Chinese belief is that a coward's liver is bloodless, hence white.—Ed.)

even of notorious bandits who have been decapitated for their crimes, and eat them with the same object in view.

Chinese science regards man as a little universe, and the human body as composed of five elements—fire, water, metal, wood and earth connected in a mysterious manner with five solid viscera, five plants, five tastes, five metals, and five colors. Diseases are regarded as being due to a derangement in the balance of the five elements, and successful medical treatment consists in restoring their equipoise.

There are beside certain mysterious dual powers, sometimes called the male and female elements in nature the yin and yang, represented under a double comma-figure familiar to all travelers in China and Korea, which pervade animate and inanimate nature, as strength and weakness, earth and heaven, light and darkness, on which medicines are believed to have a corrective and ofttimes a powerful influence. These interlocked commas are used on terminal tiles, priestly vestments, temple drums and other sacred instruments, and on much besides.

Chinese doctors are ignorant of the manner of the circulation of the blood, of the function of the heart, and of the change which the blood undergoes in the lungs; and some of their diagrams represent tubes taking their rise in the fingers and toes, and running up into the trunk, where they are either lost, or wander aimlessly about till they find their way into one of the larger organs. Though ignorant of the circulation of the blood, they believe that it is necessary fot life that the whole body should be irrigated with it.

Chinese doctors state that the whole superstructure of medical science depends upon having a correct theory of the pulse, yet they know not what causes pulsation. They believe that man has twelve pulses, corresponding to twelve organs of the body, two of which have no existence. Three of these pulses are situated above, and three below each wrist; and it is necessary for ascertaining the nature and result of disease (diagnosis resting solely on the study of the pulse) to feel both wrists; and the Chinese physician lacks words to express his astonishment at the foreign physician, who is so ignorant of the first principles of his profession as to feel only one. The rules for this important operation (translated by Dr. Hobson) are many and elaborate and the foundations on which they rest indicate their value.

Each season of the year has its proper pulse. In the first and second moons the pulse of the liver, answering to wood is "long and tremulous"; in the fourth and fifth the pulse of the heart corresponding to fire, is "overflowing;" and in the third, sixth, ninth and twelfth, the pulse of the stomach, which answers to earth, should be "slow and full." Metals govern the seventh and eight moons, and the pulse of the lungs, which answers to them, is "slendor, superficial, short and sharp?" In the tenth and eleventh moons, water reigns, and the pulse of the kidneys, corresponding thereto, is "deep and slender."

An important axiom on the pulse is: "In Spring to have the pulse of the lungs is mortal," the pulse of the heart being set aside, "for the heart is the son of the liver, which has the kidneys for its mother and the stomach for its wife."

The above is the gist of the statements regarding the pulse proper to the different seasons, due regard being had to the opposition or coalition of the five elements.

The Chinese standard "Materia Medica" fills several volumes and is of considerable antiquity. Chemistry is a science being unknown, there are few mineral preparations, and these are in crude forms, the artificial salts being unknown.

Chinese medicines are usually the vegetable productions of China and the adjacent countries, specially Tibet, while Korea provides the tonic root ginseng, to which nearly miraculous virtues are attributed, but which is so expensive that its use is confined to the rich.

Among the least objectionable of the animal substances in the pharmacopeia are the powdered blood, eyes and bones of tigers; powdered rhinoceros' horns, dried silkworm moth, red spotted lizard, stags' horns in the velvet, asses glue, human milk, extract of stags' horns, dog's flesh, bones and teeth of the dragon, dried leech, dried and powdered scorpion, dried earth worm, a preparation from toads, dried cicada and centipede, shell snake skins, bears' gall, dried silkworm chrysalis, powdered ivory, shavings of antelope horns, and the like.

Before expressing disgust, let us bear in mind that in this country the thyroid gland of a sheep is now used in medicine, and that substances as objectionable as those on this list are now used in inoculations.

Dr. Hobson, to whose researches I am much indebted, says that in a popular abridgment of this work four hundred and forty-two medicines are described, with their properties, uses and doses, all methodically classified, many of the substances being appropriate and useful, while many are worthless and disgusting. Dr. Hobson considers the plan of the therapeutic arrangement in putting the medicinal uses of drugs before their mere physical properties, worthy of commendation.

Many of the herbs used have a very definite value, but being carelessly kept, and their decoctions enormously diluted, they are not as efficacious as they might be.

Chinese doctors administer their medicines in the form of boluses, the size of a walnut, which, however nauseous, are well masticated before being swallowed; powders and decoctions, the latter frequently in doses of a pint at a time, three and four times daily, quantity being always demanded by the patient. Hence, a man to whom I gave ten five grain tabloids of quinine, to last five days, took them all at once, breaking a tooth in masticating them, and returned the next day, reeling, deaf, and in a high fever. He had understood what I said, but thought that if one tabloid was good, ten were better.

The Chinese physician has no emetics, except substances which produce unutterable disgust, hence the difficulty of dealing with opium poisoning, and the rush to a foreign doctor if there be one.

Red sulphuret of mercury has been shown by the researches of Dr. Edkins to have been regarded since the fourth century A. D., as the philosopher's stone, not only as transmuting the base metals into gold, but when absorbed into the system, as conferring immunity from death; nor does its failure in both cases shake the popular belief in the discovery of the early alchemists. Modes of rejuvenation are not confined to the use of this drug, for it is generally believed that old people can renew their youth and prolong their lives for a century by drinking human milk, which is sold at a very high price.

Surgery, properly so-called, may be said not to exist, and it is not our medicine but our surgery which wins for our doctors the high place which they occupy in China. The ignorance of anatomy is a bar to the use of the knife, and in the case of broken bones or dislocations the doctor is not thought of. Fellow workmen use their own rough methods with a dislocation. I have seen seven men hand-in-hand attempting to pull a dislocated shoulder into place, the victim being tied to a tree. In cases of broken bones, if the fracture be of a lower limb, the patient is laid on a bed, and the bone unites or remains apart, as the case may be, the ends resting against or receding from each other. Few men who break their legs can do more than limp or crawl afterward. On one occasion, however, I saw a rude splint applied to an arm, a stout, non-padded piece of bark bound on very tightly with coarse string, which had cut into the flesh, producing deep sores. The arm was livid and blackened and gangrene had already begun. No bandages are used to support limbs, nor is sticking plaster of any kind applied to bring the edges of incised wounds together. Amputation, except in the common form of decapitation, is not practised.

All kinds of tumors are mixed up together. Those which are external are "strangled," cauterised, or "needled," and those which are internal are happily let alone. There is no distinction between simple and malignant growths, and tumors are further confounded with abscesses, which are not opened, and if they break of themselves, the great idea is to close the outlet for their contents with a thick, black, resinous plaister, air proof and water proof. Plaisters are largely used, especially patches of a green one on the face or head, applied for headache, toothache, sore eyes, earache, and the like.

In the same unscientific manner all eye diseases are mixed up in hopeless confusion, and the plaisters which are applied to diseased eyes are simply abominable.

Acupuncture and the use of the cautery are the chief surgical operations, and undeniably are at times of great benefit, specially the cautery when applied as the well-known moxa, the burning of small cones of wool on the flesh. This is frequently of great benefit in rheumatism. Acupuncture, as practised by the Chinese, is barbarous. The needle, as I have seen it in the hands of the native doctors, is long and thick, something like a stiletto, often both rusty and uncleanly, and taken bare from the operator's pocket from among débris of tobacco, paper, and the like. It is thrust into an inflamed joint, or into the stomach or liver, and is mercilessly worked about, at times causing death, and often agonising suffering from increased inflammation, and in the case of a joint, frequently producing "stiff joint" or making the victim a cripple for life. Yet this is a popular practice.

Another counter-irritant is the "medicinal nail," made by mixing corrosive sublimate, arsenic and salt with gluten, letting the "nails" dry, and forcing them into the flesh, inflicting severe wounds. A modification of "needling" is used in general rheumatism. The body is stuck over with large coarse needles, with tow dipped in oil wrapped round their heads, which is set fire, and acts as a sort of cautery, producing a wound which is further stimulated by the application of the "medicinal nail."

Vaccination is now very largely practised and many benevolent people provide it freely for all who apply for it. It is customary to insert the vaccine nymph in the nostril. Some Chinese devote themselves exclusively to vaccinating and the government encourages it, but it is not taken up generally by the "medical profession"—at least in the interior. The mortality in maternity is believed to exceed 20 per cent., and some of the practises are ignorant and barbarous, but such cases are chiefly in women's hands. Many of the methods of treatment when death is approaching are, to our thinking, very objectionable; as, for example, in order to find what a patient's chance for life is, a needle is plunged into the flesh, and, if blood does not follow its withdrawal, the patient will die.

It must be understood that many Chinese customs vary in different provinces, and that, regarding some at least, it would be impossible to make a statement which would apply with equal truth to all parts of the Empire.

There are varieties in practitioners in China, as well as in fees. There is the fashionable city physician and there is the coolie, who buys a few foreign drugs, and tramps through the country professing to work cures with them. As there is no special education, so there are no restrictions. There are fashionable doctors in the cities who receive crowds of patients until a late breakfast hour, and spend the remainder of the day in visiting the sick at their own homes, taking them in the order in which their names have been entered on the visiting tablet at the door.

A fashionable doctor is carried in a sedan chair by three or four bearers, who simulate great haste. He wears a most oracular expression, and is apparently always absorbed in consulting books or notes. To save time in narrow and crowded streets, a copy of his own sign board is placed outside the patient's house, so that the bearers find it without delay. He is received with much ceremony. If the patient be a lady a bamboo screen conceals her from him. After a prolonged examination of the twelve pulses has acquainted him with all he requires to know, he writes a prescription. Some rich men require a statement of the nature of the disease, and the treatment to be pursued, for the use of the family; but this involves an additional fee, and usually a verbal statement is sufficient.

It is here that the medical humbug has an admirable opportunity for imposition. He exaggerates the seriousness of the malady and the length of time to be occupied in curing it, and by the use of abstruse scientific terms can describe with an air at once so learned and oracular as to win general belief in his power over disease. On his departure he receives his fee, called "golden thanks," wrapped up in red paper. In the case of a doctor of this type, it may be from two up to four shillings, the written opinion being two shillings more. The chair bearers are always extra.

It is not "good form" for doctors to make up their own prescriptions, but it is done by the lower classes in the profession, men who receive from sixpence to a shilling a visit.

The doctor may not pay a second visit unless he is sent for. In many cases, however, he contracts to make a cure and receives half the sum agreed upon in advance. It is a matter of usual occurrence when a patient is not cured by the first prescription to call in another doctor, and so on till four or five have been tried without a satisfactory result, when recourse is had to a god possessed of remarkable powers of healing, or to a sorcerer.

In cases of hysteria or mental derangement, the sorcerers or witch doctors are consulted; and it must be remembered that (as is generally the case in the East) throughout China, except among the educated men of the cities, there is an underlying belief that all illness is the result of the entrance of a foul demon into the body, and the last resort is the sorcerer, or the Tavist priest with his incantations.

My experience with a Chinese doctor was a mild and fairly satisfactory one. While traveling in great heat I was bitten on the foot by a large centipede and swelling, pain, and inflammation were the result, quite stopping my progress. The doctor arrived attended by a coolie, who deposited on the floor a formidable-looking chest in compartments, containing drugs, scales, weights and instruments, rather rusty, for acupuncture and cautery. After the usual formal compliments, I explained the cause of my suffering, but apparently to deaf ears. The doctor looked alert and sapient, felt my twelve pulses, above and below each wrist and back again, with a look of profound enlightenment, asked no questions, and after forty minutes spent on the pulse, proceeded to weigh and boil certain drugs over a charcoal brazier, the result being a liberal quart of a red, turbid infusion, which he directed me to take at one dose. I pointed to my foot lying on a basket under a handkerchief, and, as I thought, rather a pitiable spectacle and displaying its inflamed condition again, asked for a lotion. He barely deigned to glance at it, but compounded something, of which poppy heads were an ingredient, which at once proved beneficial.

I showed him my clinical thermometer, and made an attempt to explain its use; and he took it, tapped it, held it up to the light, and finally asked if it were for use in foreign magic. I showed him also a medicine box containing a dozen different invaluable tabloid medicines, and after considering them attentively for some time, and getting the names of some of them from my interpreter, he said they were "charms,"—that it was impossible for medicines to be in such small bulk, and persisted in his assertion. However, he asked me for some morphia tabloids, and doubtless saw reason to change his opinion.

Various odd ideas as to the structure of the human body have held sway for two thousand years, such as that the pit of the stomach is the seat of the breath as well as of the emotions of joy and delight; that thoughts proceed from the heart, as well as tubes of dubious purpose which merge themselves in the spleen liver and kidneys; that schemes originate in the liver (the residence of the soul); that the skull, pelvis, leg, and forearm are each single bones; with many other scientific beliefs. Throughout the system, disease is regarded as the product of a disagreement between the universal forces of yin and yang, or it is the direct agency of evil spirits. The dual theory being held by "regular practitioners" and vagabond quacks alike, the diagnosis is on those lines, it and the dose being administered together, the one being required to produce faith in the other—rather a workable plan.

It must be said that in some things treatment by Chinese doctors is in advance of their theories, or rather ignores them; that they can give sensible directions regarding diet, exercise, and the external treatment of some maladies, based on their observation of cause and effect; and that some of their herbal remedies, if their virtues were not drowned in water, would be valuable: It may also be said of acupuncture, that while in hundreds of cases it is so fatal to locomotion, and in some to life, there are others in which it is useful, and there would be more if it were not practised with such very dirty needles. Of cauteries and caustics little that is favorable can be said. Their use frequently entails great and prolonged suffering, generally destroying large areas of tissue, and turning trifling wounds into deep sores which discharge inwardly, owing to the abominable practice of sealing up wounds, ulcers and abscesses with an impermeable plaister. During the war I saw very many instances of the evils of this practice, where gunshot wounds had their margins severely cauterised, and then been hermetically sealed, with the bullet still inside them.

I understand that there are some sensible treatises on midwifery, embodying the results of experience; but in difficult cases, in which the skill of foreign medical women has been resorted to, malpractices of barbarous and unspeakable ignorance have been discovered. Superstitions and extraordinary methods of increasing the beneficial effect of drugs prevail generally, but they vary greatly and to describe those of Manchuria would not be to describe those of Szechuan or Kwantung.

The late Dr. Henderson, of Shanghai, in a careful study of the "Medicine and Medical Practice of the Chinese," writes thus strongly of Chinese doctors:

They sacrifice unscrupulously not only truth, but also intelligibility and reason. What excites our surprise is that century after century, and generation after generation, should have passed away in a vast country like this, and especially among such a large number of men who set themselves up as authorities and teachers of others, and that there should not be found one with a mind independent enough to make fresh observations, or sceptical enough to doubt the numerous unreasonable assertions of his predecessors. . . . Everything is false because it rests on a false foundation—falsehood is at the root of every system, rendering correct information and reliable facts impossible. What is still worse is a system which every Chinese physician knows to be utterly false, but which must be respected because it is old, and because being false it is more in unison with the other systems and institutions of China.

Dr. Wells Williams, who cannot be suspected of undervaluing anything Chinese, writes of Chinese scientists:

They have never pursued a single subject in a way to lead them to a right understanding of it.

And with this damnatory remark, I will dismiss the subject, believing that the light of western science in the region of medicine is very slowly and partially, but in the long run surely, beginning to permeate the thick darkness of the empire.

